# Software-aided automatic laser optoporation and transfection of cells

**DOI:** 10.1038/srep11185

**Published:** 2015-06-08

**Authors:** Hans Georg Breunig, Aisada Uchugonova, Ana Batista, Karsten König

**Affiliations:** 1 Saarland University, Faculty of Mechatronics and Physics, Department of Biophotonics and Laser Technology, Campus A5.1, 66123 Saarbrücken, Germany; 2 JenLab GmbH, Schillerstr. 1, 07745 Jena, Germany and Science Park 2, Campus D1.2, 66123 Saarbrücken, Germany

## Abstract

Optoporation, the permeabilization of a cell membrane by laser pulses, has emerged as a powerful non-invasive and highly efficient technique to induce transfection of cells. However, the usual tedious manual targeting of individual cells significantly limits the addressable cell number. To overcome this limitation, we present an experimental setup with custom-made software control, for computer-automated cell optoporation. The software evaluates the image contrast of cell contours, automatically designates cell locations for laser illumination, centres those locations in the laser focus, and executes the illumination. By software-controlled meandering of the sample stage, in principle all cells in a typical cell culture dish can be targeted without further user interaction. The automation allows for a significant increase in the number of treatable cells compared to a manual approach. For a laser illumination duration of 100 ms, 7-8 positions on different cells can be targeted every second inside the area of the microscope field of view. The experimental capabilities of the setup are illustrated in experiments with Chinese hamster ovary cells. Furthermore, the influence of laser power is discussed, with mention on post-treatment cell survival and optoporation-efficiency rates.

Introducing foreign (genetic) material into targeted cells has become an indispensable technique in biomedical research^1^,^2^,^3^. Particular interesting is the possibility to transfect cells, i.e. directly re-program one cell type into another. This has, for example, enabled the ground-breaking artificial creation of induced pluripotent stem cells (iPS) which have the potential to differentiate into all body-cell types and thereby offer the prospect of using them in cell-replacement therapies for several illnesses^4^,^5^. To transfect a cell requires permeabilizing the membrane, which is normally impenetrable for large molecules. Common approaches rely on the use of chemical delivery vehicles, viral vectors, direct microinjection, or cell-membrane permeabilization through electrical pulses or sound waves^6^,^7^,^8^. In contrast to these methods which aim at global cell populations, laser-assisted cell-membrane poration (“optoporation”), i.e. to use focused laser pulses to transiently perforate the cell membrane, targets cells individually^9^,^10^,^11^,^12^,^13^,^14^,^15^,^16^,^17^. This approach offers several advantages: it is highly selective, efficient, reproducible, non-contact, aseptic, compatible with standard microscope optics, little dependent on cell type and condition as well as easier to perform than most other techniques like e.g. microinjection^2^,^16^,^18^. Optoporation has been realized using a wide range of experimental parameters, e.g. with laser-pulse durations ranging from ns to sub-15 fs, with illumination by single up to several million pulses per cell, and over a large range of pulse energies (pJ to several tens of μJ), illumination times (few to hundreds of ms), as well as a range of focusing conditions by using different numerical aperture objectives and focal-volume shapes^9^,^16^,^17^. Several authors have reported that particularly ultrashort pulses, which can induce a tiny, transient sub-micrometre pore in the cell membrane allowing for the penetration of substances by diffusion, are conducive to high post-treatment cell survival and optoporation efficiency^10^,^11^,^12^,^13^,^18^,^19^. The often envisioned automation of the process, however, is difficult. Cells usually have to be manually addressed in a procedure normally consisting of (i) preparing the cells in a suspension that contains the molecules which are intended to enter the cells, (ii) locating the cells of interest, (iii) identifying a suitable spot to apply the laser so as to generate a hole in the cell membrane (iv) either firing the laser onto that particular spot by guiding the beam with mirrors, suitable beam shaping or by centring the spot by appropriately moving the microscope stage onto the laser focus position which is kept fixed and (v) observing cell changes at various later times^16^. This process is tedious, time-consuming and while enabling to address individual cells which make single-cell studies possible, only a small number of cells can actually be targeted per procedure. The number of addressable cells for manual laser optoporation reported with different setups varies approximately between 20 and about 1000 cells per hour^9^,^17^,^20^. Another hurdle posed for automating the procedure is the necessary optimization of several parameters such as exposure time, laser power and optimal location of irradiation for specific cell lines or experimental setups which can be time consuming as well and may have to be repeated between experiments^15^. A software-aided automation of laser optoporation is elaborated herein so as to significantly increase the number of treatable cells and in doing so, simplifying the whole process for the user. Software to analyse and quantify the outcome of the optoporation experiments, in terms of post-treatment cell viability, as well as optoporation efficiency has also been developed. This is desirable not only to facilitate and speed-up the optoporation procedure but to be able to efficiently and reproducible explore the effects of different amounts and types of foreign genetic material, and the optimization of further experimental parameters. The goal of automating the process makes it necessary to apply a software tool which quickly identifies and selects suitable cell positions for laser illumination and controls the relevant hardware. An automation based on a sophisticated analysis of the cell morphology as described in Ref. 20 can by itself be very time consuming due to the many possible eventualities that may occur with different cell types, individual cell variations as well as varying illumination conditions and may ultimately offer little to no real net time benefit compared to a manual approach. Therefore, a simple yet rapid cell-detection algorithm based on the image analysis of contrast variations was implemented to identify cell positions suitable for transient laser illumination and combined with microscope hardware control. It was found that with an illumination duration of 100 ms, an average of 7-8 different positions can be illuminated per second for a typical cell confluence. Our experimental setup employs a broadband fs laser, however, the software control and analysis acts independently from the particular laser source and experimental setup and could therefore be straightforward combined with other light sources. The experiments were carried out with Chinese hamster ovary (CHO) cells, a commonly used model cell type for biological research and transfection experiments.

## Materials and Method

### Experimental Setup

**Figure 1:**
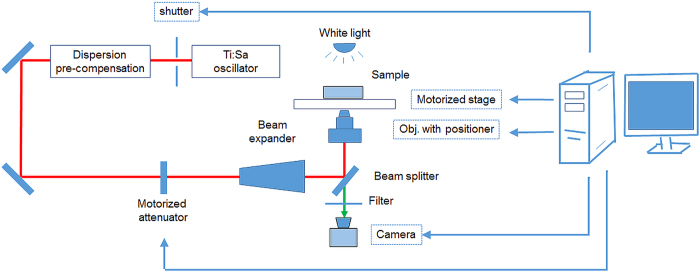
Scheme of experimental setup for software-aided automatic cell optoporation. Cell positions in a culture dish placed on the microscope stage can be identified, centred and laser illuminated by custom-made software. The arrow lines indicate the computer control of the corresponding hardware.

The experimental system for software-aided automatic cell optoporation is schematically shown in Fig. 1. It consists of a microscope (Zeiss Axio Observer, Zeiss) which has been transformed into a multiphoton imaging system (Femtogene, JenLab GmbH) and a Ti:sapphire oscillator. The multiphoton imaging system with a common laser-scanning setup, i.e. galvano scanning mirrors, a beam expander, dichroic mirror, and a microscope objective, was described previously in detail^21^. The Ti:sapphire oscillator (Integral, FemtoLasers GmbH) generated pulses with a centre wavelength of about 800 nm at a repetition rate of 85 MHz and about 420 mW mean power. Chirped mirrors were used to pre-compensate for the group-velocity dispersion of the optics so that pulses with durations of sub-15 fs were present behind the objective which had been confirmed by autocorrelation measurements^21^. For the optoporation experiments described here, the multiphoton imaging capability of the system was not used and the laser-scanning mirrors were kept at fixed positions. Glass-bottom dishes containing the cell cultures were placed on a motorized x-y-microscope stage (Märzhäuser Wetzlar, SCAN IM 130 × 100 with a ball screw pitch of 1 mm). A 1.3NA objective (40X, Neofluar, Zeiss) was used to focus the laser pulses. The vertical position of the objective was controlled by a built-in piezoelectric positioning system (MIPOS 100, Piezosystem Jena GmbH, Germany). The laser power at the sample was controlled through the rotation of a Glan laser polarizer inside the beam path. The duration of laser illumination was selected by setting the open duration of a fast mechanical shutter (Thorlabs). Images were recorded with a 1.3-megapixel microscope camera (SPOT idea, SPOT Imaging Solutions) through the same objective used for focusing the laser pulses. The microscope stage, camera, piezoelectric positioning system and the optical beam shutter were all controlled by custom-made LabView-based software which is described in more detail in the discussion section. The computer that was used had a CPU with Intel core 2 duo E8400 processors (3 GHz) with 2 GB RAM and on-board integrated video card.

### Sample Preparation

CHO-K1 cells were cultured in 35-mm glass-bottom dishes with 500 μm grids (IBIDI GmbH, Martinsried, Germany) in 1.5 ml of F-12 nutrient mix (#21765–029, Gibco® by Life Technologies) supplemented with 10% fetal bovine serum (#10270–106, Gibco® by Life Technologies) and 1% Penicillin-streptomycin (#P4333, Sigma-Aldrich) to sub-confluent monolayers. Afterwards, the cells were incubated at 37 °C with 5% CO_2_ for approximately 24 hours. Prior to the optoporation, the cells were washed once with Dulbecco’s phosphate buffered saline (D-PBS, #H15-002, PAA Laboratories GmbH) and bathed in 1 ml of calcein AM solution (ratio 1:2 as provided in producer´s protocol; #R37601, Molecular Probes® by Life Technologies) in OptiMEM (#11058-021, Gibco® by Life Technologies) as well as 4′,6-Diamidin-2-phenylindol (DAPI, ratio 1:60, #D9542, Sigma-Aldrich). After the optoporation, cells were washed with D-PBS, bathed in a 300 μl solution of calcein AM in OptiMEM (ratio 1:2) and placed in the incubator for approximately 30 min. Subsequently, 1 ml of a solution of ethidium bromide in culture medium with a concentration of 0.15 μl/ml (EtBr; #E1510, Sigma-Aldrich) was added to the bathing solution and the cells were incubated for 1–2 min at room temperature. For the transfection experiments, a pAcGFP1-Mem (Clontech, #632491) plasmid vector was added to the cell suspension (8 μg/ml) shortly before optoporation. After treating the cells, they were washed once with D-PBS, placed into the incubator kept at 37 °C in 5% CO_2_ atmosphere and daily inspected for green fluorescent protein (GFP) fluorescence signals with an inverted fluorescence microscope (Carl Zeiss AG, Oberkochen, Germany).

### Cell image segmentation and classification

**Figure 2:**
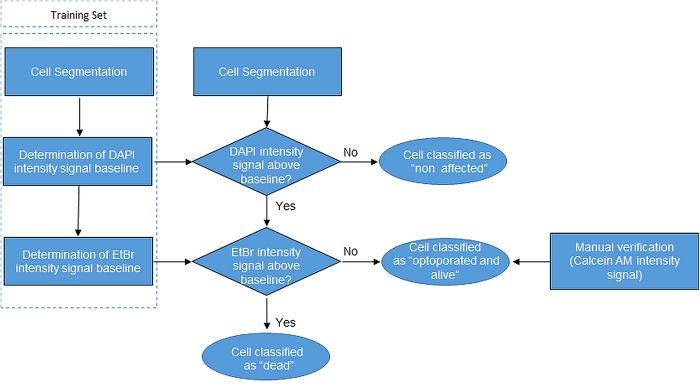
Flow diagram of two-step procedure to evaluate the outcome of the laser illumination based on DAPI, EtBr and calcein fluorescence staining. DAPI fluorescence is used for both to segment cells and evaluate an optoporation. The viability of cells with DAPI fluorescence exceeding the threshold for optoporated cells is in a second step assessed according to EtBr and calcein fluorescence intensity.

DAPI is a fluorescent dye which binds strongly to DNA in the cell nucleus^22^. In low concentrations, like the one used in these experiments, a strong fluorescence signal is only observable in cells whose membrane were perforated, i.e. in dead and/or optoporated cells, as DAPI passes very slowly through intact cellular membranes^23^. Calcein AM is originally a non-fluorescent compound which however, emits green-fluorescence after hydrolysis by intracellular esterase in living cells. EtBr is a fluorescent dye that bounds to DNA and identifies cells with disrupted membranes, i.e. dead cells. Together these fluorescent dyes provide mutually complementing information on the cell condition. To evaluate the outcome of the optoporation experiments, phase contrast and DAPI, calcein and EtBr fluorescence images of all treated and untreated control regions were recorded using an inverted Zeiss microscope (Carl Zeiss AG, Oberkochen, Germany) equipped with an LED module (Colibri.2) for fluorophore excitation. Treated and untreated control regions were in each case imaged with equal exposure time and excitation-light intensity. Cells were detected in the images and classified in a two-step procedure illustrated in the flow diagram in Fig. 2. The cell detection was done by image segmentation using grey-scale morphology of the DAPI fluorescence images. After image histogram normalization, a morphological opening operation was applied to supress small intensity peaks. Then an extended h-maxima transform was used to segment the cells^24^. After segmentation the cells were classified by comparing their fluorescence signals to a set of intensity thresholds that were obtained using a training set (control images). The DAPI fluorescence intensity threshold was defined as the average intensity value plus two standard deviations, as computed from the living control cells. First the value of the DAPI fluorescence intensity of a segmented cell was evaluated. A DAPI intensity value below threshold indicated that the cell’s membrane had not been affected by the laser illumination while an above threshold value indicated that the cell was either dead or optoporated. The subset of cells with DAPI fluorescence above the threshold, was further assessed in a second step according to their EtBr fluorescence intensity to distinguish between “dead” and “optoporated and alive” cells. While a high value EtBr intensity value indicated dead cells, the absence of any EtBr fluorescence intensity was regarded as an indication for a successful optoporation of the cell. Manual inspection of the calcein fluorescence signal intensity was additionally performed for all cells classified as “optoporated” to confirm the results by the software algorithm. The ratio of dead cells inside a region of interest (ROI) was calculated as r_d_ = N_d_/N_total_, N_d_ being the number of cells which did not show calcein but EtBr fluorescence; N_total_ being the total number of cells inside the ROI. The ratio of “optoporated and living” cells was calculated according to r_o_ = N_DAPI_and_calcein_ /N_total_, with N_DAPI_and_calcein_ being the number of cells which showed both DAPI fluorescence above the threshold and clear calcein fluorescence.

### Software for automated optoporation

**Figure 3:**
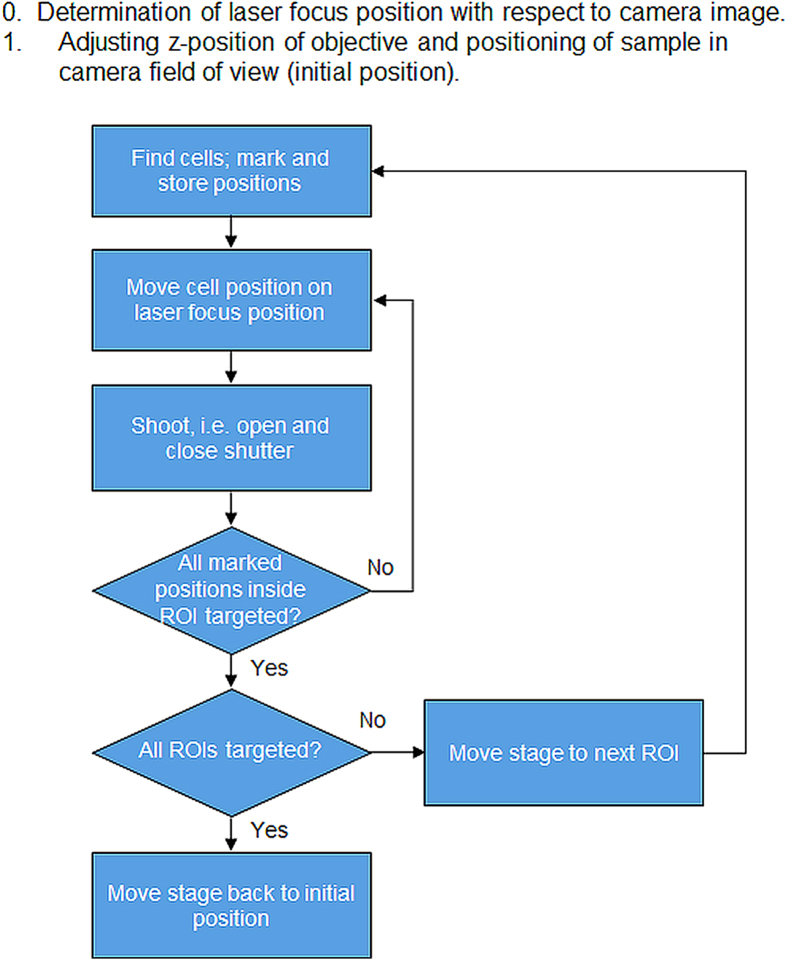
Flow diagram of custom-made program for automated transfection. Cell positions are first identified according to the image contrast in a microscope brightfield image. The positions are then consecutively centred at the laser focus and illuminated by engaging a mechanical shutter. Afterwards the next ROI is targeted until the preselected sample region is completed.

**Figure 4:**
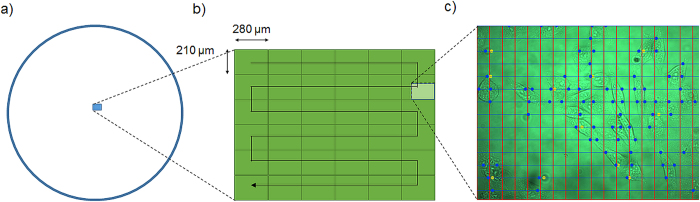
(a)Illustration of scanned area of 6 × 6 FOM region inside a 35 mm cell culture dish. **b)** Illustration of meander scanning of microscope view field. **c)** Section of single microscope field of view with raster that illustrates cell search areas. Contour contrast positions are determined along the blue lines for the sections between the red lines. The circles indicate the positions designated by the algorithm for laser illumination, i.e. the laser target positions. The very close-laying yellow circles are removed from the set of targeting positions.

**Figure 5:**
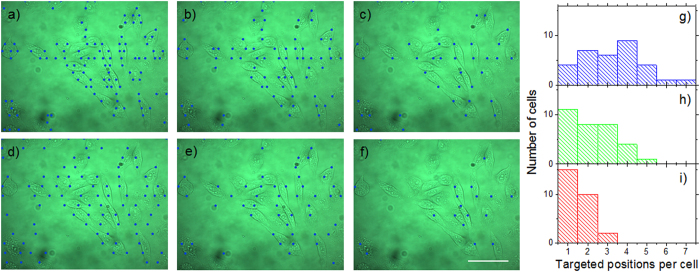
Influence of the two software parameters “search area size” and “minimum edge detection” for the cell contour recognition algorithm on the number of detected positions (blue dots) per cell . **a–c**) square search areas with edge lengths of 6 μm, 12 μm, and 18 μm, respectively. **d–f**) value of the “minimum edge strength” parameter of 5, 15, and 25, respectively. **g–i**) Distributions of the number of detected positions per cell from the images shown in Fig. 5a–c. Scale bar: 50 μm.

The software for automated cell optoporation controls several hardware components and determines the laser-illuminating positions through software-based evaluation of a microscope image of a region of the cell samples. The software is able to locate, target and laser-illuminate points on cells by determining a cell´s position, centring this position onto the laser focus and opening and closing the mechanical shutter. A scheme of the program flow is given in Fig. 3. After addressing all located cell positions inside a microscope´s field of view (FOV) the stage moves for a length corresponding to the size of the FOV in a meander shape, so that a large sample area can be covered in a mosaic pattern. Figure 4 illustrates the dimensions. Inside a 35-mm cell culture dish (Fig. 4a) 6 × 6 FOVs are scanned in a meander pattern (Fig. 4b) and bright field images recorded. In these bright-field images, positions for laser illumination are identified and designated for laser illumination (marked as blue circles in Fig. 4c). The software-based automatic identification of target positions on the cells for laser illumination is necessary for the software-assisted automated cell optoporation. The exact locations on the cell, i.e. cell membrane, cytoplasm or nucleus, as well as the number of illuminated positions per cell greatly influence optoporation as well as the post-treatment cell survival^15^. For the automated identification of laser target positions on the cells, images with a high contrast such as phase contrast images would be preferable, however, the use of a single microscope objective for laser focusing and imaging significantly impedes the application of elaborate contrast microscopy. The bright-field images of the cell samples exhibit only low image contrast to the human eye, yet still sufficient for the algorithm. To determine a position for laser targeting, the camera images are sub-divided into square ROIs with sizes that are chosen by the user to roughly correspond to the typical size of the targeted cells, e.g. lengths of 10–20 μm for CHO cells. The cells are identified by determining “image edges” along the horizontal ROI border lines with the built-in LabView function “IMAQ Rake 3” (National Instruments, LabView Vision development module). This function evaluates changes in contrast and slope to determine “edge positions” along a set of parallel lines. Edges are defined in this context as significant changes in the grayscale values between adjacent pixels. To identify an edge, the IMAQ Rake 3 function pixelwise scans the pixel profile from beginning to the end. A rising edge is identified as the first point at which the pixel value exceeds the sum of a threshold value and an internal hysteresis value. The threshold value is set by the user (“minimum edge strength”) to define the minimum edge strength to qualify as an edge. With the hysteresis value different edge strengths for the rising and falling edges can be declared. Furthermore, the algorithm can also use a kernel operator to compute the edge strength based in a local approximation taking into account the several adjacent pixel values. Further information on the edge concept in NI Vision and the “IMAQ Rake 3” function is given in Ref. 25. Figure 4(c) illustrates the implemented procedure. “Edges”, marked by filled circles, are identified along the blue horizontal lines between the neighbouring vertical red lines. These filled circles mark the automatically determined positions for intended laser illumination later. In case two target positions lie very close to each other one of them is removed from the set of illumination positions by the software (yellow circles), to minimize the stress on the cells due the laser-illuminating. The separation (“search area size”) between the horizontal blue lines can be fine-tuned by the user to accommodate different cells sizes and influence the average number of shots per cell. Figure 5a)–c) illustrates the effect of that parameter. The smaller the distance the more positions are found per cell. The number of target positions on one cell is in this way chosen indirectly by changing the parameter “search area size” and has to be selected in a trade-off between cell survival and optoporation efficiency (the more shots on a cell the more likely it will not survive the procedure but the higher the chance for optoporation) before starting the optoporation procedure. In the example exhibited in Fig. 5a)–c), separation values of 6 μm, 12 μm and 18 μm result in the distributions of the number of detected positions for each cell shown in Fig. 5g)–i). On average 3.3, 2.3 and 1.5 positions were allocated on each cell in Fig 5a)–c), respectively. A separation value that exceeds a typical size of a cell leads to cells being overlooked. Another parameter that affects the finding of the allocated position is the “minimum edge strength” which is an input parameter used for the edge recognition function. Its influence is illustrated in Fig. 5d)–f) with the values of 5, 15, and 25, respectively, and with a fixed ROI length of 12 μm. In this example, 97, 54 and 19 positions are found, respectively. For a “minimum edge strength” value of 5 (Fig. 5d), many “edge” positions in the image are found which actually do not belong to cells but are merely image artefacts. With a “minimum edge strength” value of 25, many cells are not detected (Fig. 5f). Usually, for one set of experimental conditions (cell shape, illumination conditions) the input parameters have to be adjusted for that specific procedure. By visual inspection of the identified cell positions which are shown in the still image of the software interface, the user can change and select suitable parameters such that cell positions are identified and that on average a desired number of positions per cell are found. A prerequisite for the algorithm to work properly are adherent cells in a monolayer. The density and type of cells does not affect the principle operation of the procedure as long as the cells are visible in the bright field image, i.e. as long as detectable contrast changes are present. Detection of cells in regions with different cell densities is exemplified in the supplementary material (movie 1).

It would be possible to apply a more elaborate cell-recognition algorithm that, for example, determines the entire cell shape and marks precisely the desired number of target positions on that cell as described in Ref. 20. However, concerning how different samples should be treated, there can be numerous variables and inconsistences as a result of different cell types, shapes as well as confluence and illumination conditions; making such an algorithm inherently complex and time consuming. The analysis time needed for the image processing algorithm described in Ref. 20 as being between 5 and 40 min per image is too long for practical experiments as such a prolonged exposure to the environmental conditions in the laboratory (temperature, humidity, CO_2_) affects cell viability and contradicts the intention of speeding up the optoporation procedure. It was found that the fast and easy-to-implement contour determination is sufficient for addressing a large number of cells with numerous possible shape distributions. The time necessary for identifying and marking the cell positions in Fig. 5, for example, is less than 40 ms. The time taken to determine the target positions is negligibly short when compared to the time required to move the microscope stage and to open and close the laser shutter. The employed microscope stage requires approximately 15 ms for a position change of 10 μm, including the time needed to accelerate, move and stop; all of which needs to be added to the actual illumination time. In a practical cell experiment with a laser illumination time of 100 ms, 7–8 positions could be targeted per second. This implies that centring a position on the laser focus by the microscope stage took about 30–40 ms per position for a typical cell confluence.

As a prerequisite for the experiments, the position of the laser focus inside to the camera image needs to be defined by focusing the laser on a fluorescent material and determine the pixel position of the corresponding fluorescence signal. Following this, the cell culture sample is positioned on the microscope stage and the focus height is manually optimised until a clear image is visible or by test illumination of a few cell positions while changing the objective height until the generation of transient gas bubbles formed on cellular membrane is visible which can be used as a measure for the optimal height^16^. The optimal height may differ slightly for different microscope stage positions due to a possible tilt of the cell culture dishes. With a sufficient number of correct x-y-z values a height correction depending on the exact stage position can be interpolated from plane fitting those points.

**Figure 6:**
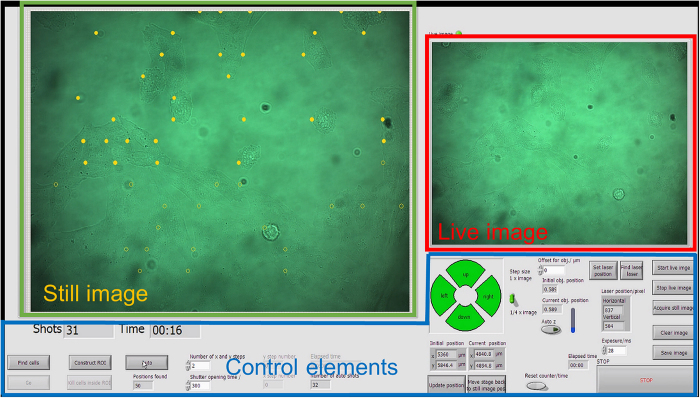
Overview of the software interface. The interface consists of three main parts: a still camera image, a live camera image and a control area for user input and parameter display. The camera shows bright field microscope FOVs. The still image shows the FOV which is in the overall procedure scanned across the sample in a meander shape. Within each still image a set of cell positions is detected which are subsequently laser illuminated by centring these positions at the laser focus and synchronized opening and closing of a mechanical shutter in the laser beam path. The laser focus is always kept fixed while a microscope stage moves the sample. Detected but not yet illuminated cell positions are indicated by open circles, laser-illuminated positions are marked by filled circles. The still image does not change during the illumination procedure of cells in the particular FOV region. The actual camera image is shown in the live image. In the control part of the interface the user can start the optoporation procedure, move the stage and enter program parameters. A movie of the software in operation is available in the supplementary material.

An overview of the software interface is given in Fig. 6. The interface comprises of windows for the microscopic still image which shows the sample FOV as well as a live image window of the same area, including several control buttons.

### Cell-optoporation and transfection experiments

**Figure 7:**
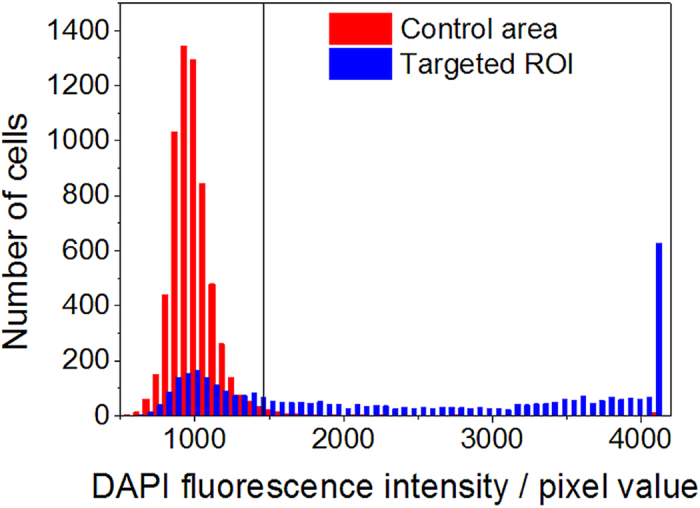
Histograms of DAPI fluorescence intensities for laser illuminated (targeted) and control (not illuminated) cells. The vertical line indicates the threshold above which cells were counted as “optoporated”. The threshold is the average fluorescence value of the set of control cells plus two standard deviations determined from fitting a Gaussian to the intensity distribution.

**Figure 8:**
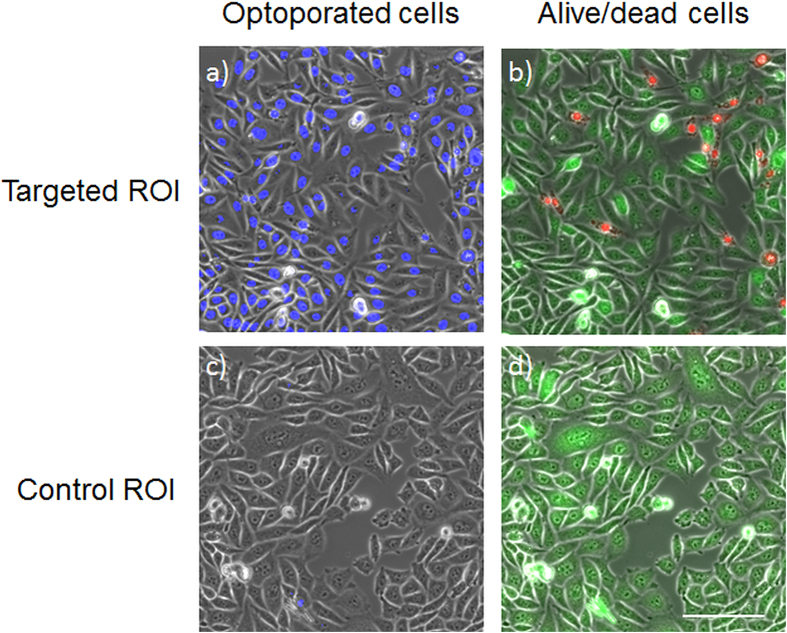
Microscope images of representative selections of a laser illuminated (targeted) ROI (**a**, **b**) and a control ROI (**c**, **d**) after fluorescence staining. **a, c**) Overlay of phase-contrast and above-threshold DAPI fluorescence (blue, optoporated cells) images. **b, d**) Overlay of phase contrast, EtBr (red, dead cells) and calcein (green, alive cells) fluorescence intensity images. Scale bar: 100 μm.

**Figure 9:**
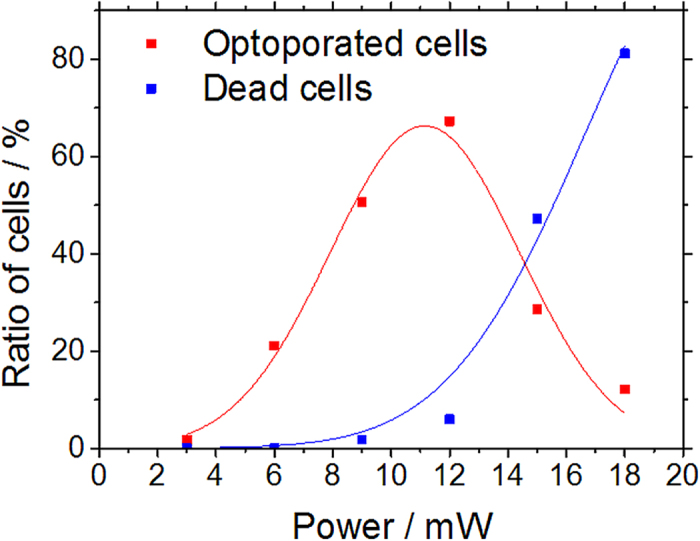
Ratio of dead living optoporated cells for different laser powers of all cells in the ROI. The illumination time was kept fixed at 100 ms. Each data point represents 400–700 cells. The lines are guides to the eye only.

Figure 7 shows histograms of the DAPI fluorescence of 3609 targeted and 6327 control cells in an optoporation experiment with CHO cells. The parameters of the cell-detection algorithm were chosen such that about two to four positions were targeted per cell. The targeted sample region had a size of about 400000 μm^2^. The optoporation was evaluated applying the procedure described above, i.e. by fluorescence staining of the cells after the procedure with DAPI, EtBr and calcein and software evaluation of the fluorescence images of the targeted region. A clear accumulation of DAPI fluorescence intensity values around 1000 is visible in Fig. 7 for the control cells while the intensity of many illuminated cells clearly exceeds that number. The fluorescence intensity of both control cells and targeted cells was recorded with the same exposure time which was chosen according to the brightness of the control cells. Several of the targeted cells even show a fluorescence intensity which saturated the 12-bit camera. These cells caused the peak in the highest histogram interval. The spreading of the DAPI fluorescence intensity above the threshold value is most likely due to a different number of illumination positions on each cell (two to three) and the influence of the actual targeting location within a cell which may influence the penetration rate of DAPI^15^. According to the classification “optoporated and alive” cells exhibit significant DAPI and calcein but no EtBr fluorescence. This is illustrated in Fig. 8 which exhibits overlays of phase contrast and fluorescence images of DAPI (Fig. 4a–c) and combined calcein and EtBr (Fig. 8b–d) fluorescence, respectively. Figure 9 shows post-treatmentcell survival rates and ratios of successfully optoporated cells from an experimental series using the described software with different laser powers and a fixed illumination time of 100 ms. The 100 ms-illumination time and power range were selected based on laser parameters described in Ref. 11. Each data point in Fig. 9 represents about four to seven hundred cells. For small laser powers the ratio of dead and optoporated cells was similar to the corresponding number in the control samples, i.e. below 0.1%. For higher laser powers this number decreased since more cells died due to the laser illumination. At 18 mW the ratio of dead cells reached 80%. This increase results from the rise of irreparable cell damage with increasing laser power. The ratio of optoporated and living cells clearly goes through a maximum with about 67% at a laser power of 12 mW with increasing laser power. The cell ratios are based on accounting for all cells of the targeted area, i.e. cells missed by the algorithm were included. All cells were laser illuminated in less than 30 min. Most of the time was consumed by adjusting and confirming the optimal objective height which may differ for very large ROI, i.e. for different positions of the sample due to a small horizontal tilt of the culture dish. In several runs we found that effectively about 10,000 cells can be laser illuminated in one hour. The manual adjusting could be reduced or completely avoided by using a more precisely aligned sample holder, however, at the cost of more elaborate sample positioning. A movie exemplifying automated laser-illumination is available in the supplementary section (movie 1). The movie shows the still image of the software interface for a 15-minute-long operation (first 30 s in real time and the rest in time lapse). It illustrates the identification of cell positions (marked by empty circles), the subsequent movement of the stage and the laser illumination (the cell positions are then marked by filled circles). The laser illumination time per position in this example was set to 100 ms. During the 15 minutes covered in the movie 6281 cell positions were targeted and laser-illuminated. By counting a random test sample of 281 cells we found 404 illuminated positions, i.e. on average 1.44 target positions per cell, i.e. about ~4360 individual cells were laser illuminated in 15 minutes. The exact number of cells that can be laser illuminated in a given sample depends on (i) the cell density, (ii) the time to identify cell positions (computer hardware), (iii) the stage movement speed, (iv) the selected illumination time, and (v) the camera exposure time for the bright field image. i) The cell density affects the average traveling distance of the stage between cells as well as how many microscope FOV regions are headed for. ii) The time to identify a single cell position in an image file with our algorithm (and the computer hardware described above) is <1 ms. iii) The travel speed of the microscope stage is 10 μm in 15 ms. iv) The illumination time can be selected by the user. In Ref. 11 and 16 typical illumination times between 1 ms and 100 ms are reported. v) A bright field image of a region with an area of about 220 μm × 280 μm (“still image”) is taken for every microscope FOV of the mosaic pattern. The stage movement time to move to the next FOV position is typically about 150 ms. The required camera exposure time depends on the illumination conditions and camera type. In our case typical camera illumination times are in the range of 20–50 ms. Dominating factors for the number of cells that are treatable in a given time interval is cell density and the illumination time. For higher cell density and shorter illumination times the number of treatable cells can be even larger than in the example movie. The exact number of successfully optoporated cells further depends on the optoporation efficiency and the precise targeting in z which is currently the main limitation. Another approach to circumvent this limitation would be to use the software for height correction by generating effectively a height map of the sample as described in the previous section or by using the software to automatically laser-illuminate an identified cell position at several heights with steps of a few μm in between. Furthermore, beam shaping could be straightforward employed to axially elongate the laser focus region, e.g. by generating a quasi-Bessel beam which would relax the requirement for exact height focussing^18^.

**Figure 10:**
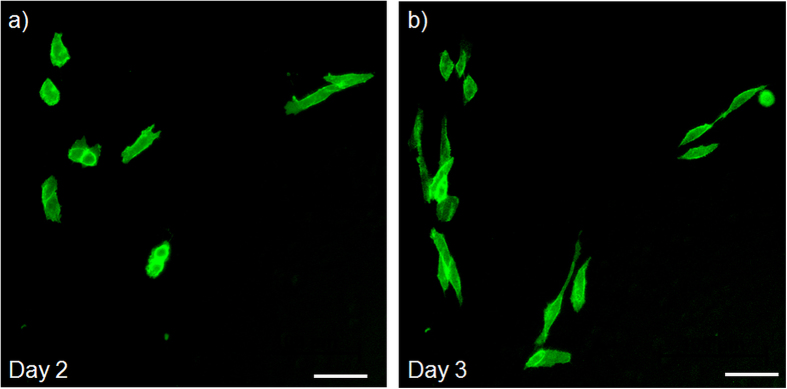
Representative fluorescence images of viable transfected CHO cells that display normal cell morphology and exhibit green GFP fluorescence two (**a**) and three (**b**) days after laser transfection with the automated software procedure, respectively. Successfully optically transfected cells exhibit green GFP fluorescence while no spontaneous transfection has been observed in controls.

Besides optoporation also successful laser-assisted transfection has been confirmed by observing GFP expression by fluorescence microscopy. Figure 10 shows representative phase contrast fluorescence images of a CHO cell sample two and three days after optoporation. For these proof of principle experiments an illumination time of 100 ms and 10 mW laser power were used. The transfected cells exhibit normal cell morphology. Spontaneous transfection of cells expressing GFP after exposure to GFP plasmid but no laser radiation is in principle possible with low probability but has not been observed in controls. The cell cultures have been observed for several days to confirm these observations.

RNA-seq generated during this study has been deposited in GEO under accession number GSE63610.

## Conclusion

An experimental setup was presented with software intended for automated cell optoporation and transfection. The software evaluates image contrast changes due to cell contours so as to determine cell locations and controls hardware to centre these positions on the laser focus and to transiently laser-illuminate these positions by engaging a mechanical shutter. Through automatic shifting of the evaluated region, in principle all cells within a culture dish can be automatically targeted. The software-based fast automation leads to a significant increase in the number of treatable cells compared to a manual approach. The time required for identifying, targeting and illuminating the cells is dominated, for durations exceeding 20 ms, primarily by the illumination duration itself. For an illumination duration of 100 ms, 7–8 cell positions can be targeted per second. Including the time for sample positioning and verification of the optimal height effectively 10,000 cells can be treated per hour without in-between objective height adjustment even more. This number could be further increased by employing measures to make the height adjustments unnecessary like elongating the focal region through beam shaping e.g. by generating a quasi-Bessel beam geometry or by amending the software to laser-illuminate the identified cell positions at several heights. The experimental capabilities were illustrated in CHO-cell experiments, and maximum optoporation rates of surviving cells were observed in the range of 60–70%.

## Additional Information

**How to cite this article**: Breunig, H. G. *et al.* Software-aided automatic laser optoporation and transfection of cells. *Sci. Rep.* **5**, 11185; doi: 10.1038/srep11185 (2015).

## Supplementary Material

Supplementary Information

Supplementary Information

Supplementary Information
