# Supplementary material for: Software-aided automatic laser optoporation and transfection of cells
Source: Sci Rep. 2015 Jun 8;5:11185. doi: 10.1038/srep11185 (PMC4459191; doi:10.1038/srep11185)
Supplement: Supplementary Information [file srep11185-s1.pdf]

Supplementary Information

Software-aided automatic laser optoporation and transfection of cells

Hans Georg Breunig[a,b]*, Aisada Uchugonova[a,b], Ana Batista[a], Karsten König[a,b]

[a]*Saarland University, Faculty of Mechatronics and Physics, Department of Biophotonics and Laser Technology, Campus A5.1, 66123 Saarbrücken, Germany*

[b]*JenLab GmbH, Schillerstr. 1, 07745 Jena, Germany and Science Park 2, Campus D1.2, 66123 Saarbrücken, Germany*

E-mail: h.breunig@blt.uni-saarland.de

Legend for Movie 1

The movie shows the still image of the software interface during an operation procedure. The software performs the following steps: 1) Recording an image from the microscope camera. 2) Identifying a set of cell positions for laser-illumination. 3) Moving of the microscope stage to center one of these positions on the laser focus. 4) Opening and closing of a mechanical shutter in the beam path to start and stop the laser illumination. 5) Centering the next cell position on the laser focus and engaging the shutter until all positions from the set have been targeted. 6) The stage is then moved to the neighboring FOV area and a new camera image is taken where again positions are identified and laser illuminated. The cell positions identified for subsequent laser-illumination are labeled with empty circles. Position that were already laser illuminated are marked with filled circles. In this example, the movie covers an operation time of 15 minutes (first 30 s are shown in real time and the time afterwards in a time lapse). The laser illumination time per position was set to 100 ms. During the 15 minutes 6281 cell positions were identified and laser-illuminated.

Legend for Movie 2

The movie shows the software interface during operation of the optoporation procedure in real time. The interface consist of three main areas: a still image on the left, a live image on the right and control and input buttons below (see also Fig. 6 and caption). The still image displays the field of view where a set of cell positions is identified. Open circles mark identified cell positions, filled circles mark identified and already laser-illuminated positions. For laser illumination, positions which are marked by open circles are centred at the laser focus and a mechanical shutter is opened and closed. The whole procedure and the hardware components are controlled by one common software. The actual movement of the stage for centring and laser illuminating of the cell positions is shown in the live image. The live image is constantly updated. The still image only changes, i.e. displays a new image captured by the microscope camera, when the stage moves to a new field-of-view area after all identified cell position in one area have been laser-illuminated. By moving from one field of view area to the next in a meander shape, cells in a large region of the sample dish can be targeted. In this example movie, the illumination

time was set to 300 ms longer than typically chosen for actual laser optoporation. Such a long illumination time was chosen for illustration purposes to slow down the overall procedure for a clearer demonstration of the operation.
